# Ionomic exploration of the geographical and geological origins of mountain pasture cow milks in the French Massif central

**DOI:** 10.1016/j.fochx.2026.103501

**Published:** 2026-01-05

**Authors:** Camille Martin, Abdelmouhcine Gannoun, Christophe Poix, Laurent Rios, Christian Coelho

**Affiliations:** a Université Clermont Auvergne, INRAE, VetAgro Sup campus agronomique de Lempdes, UMR F, F-15000 Aurillac, France; b Laboratoire Magmas et Volcans, Université Clermont Auvergne, CNRS, IRD, OPGC, Clermont-Ferrand, France

**Keywords:** Raw cow milk, Multielement concentration, ICP-MS, Traceability, Volcanic region, Geographic origin

## Abstract

Ionomic profiling was used to characterize the multielemental composition of raw cow milk during mountain pasture in the French Massif central, and to assess its potential as an indicator of geographical and geological origin. Eighteen milk samples were analyzed by ICP-MS from farms in three mountain sectors (Sancy, Cantal, Other), located on volcanic and non-volcanic sites. Of the 61 analyzed elements, 32 were consistently quantified, several of them (Pd, Zr and Nb) being reported for the first time in milk. Multivariate statistical analyses permitted to differentiate geographical provenances and geological conditions based on specific sets of elements. Pearson correlations analyses revealed geology-driven soil-to-milk transfer processes, exemplified by the element pairs - (Rb,Te) and (Sr,Pd), underscoring the influence of terroir on milk composition. Further research should focus on the soil-plant-milk continuum to better understand these geochemical pathways and to support the concept of milk terroir for the PDO cheese sector.

## Highlights

• Ionomic profiling applied to raw cow milks from French mountain pastures
• ICP-MS identified 32 elements with geographic and geologic variability.
• Elements (Pd, Zr, Nb) reported for the first time in raw cow milk.
• Multivariate analysis confirmed the relevance of geological conditions for milk traceability.
• Rb—Te and Sr—Pd revealed geology-driven soil-to-milk transfer processes.

## Graphical abstract

**Figure:**
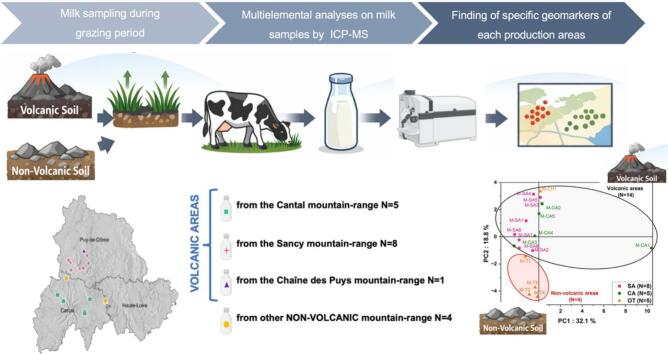

## 1 Introduction

For several decades, ICP-MS analysis of trace element concentrations in food has gained interest because of its key role in product traceability and authenticity, as well as in assessing nutritional benefits and human health risks (Drivelos & Georgiou, 2012; Luykx & van Ruth, 2008). These analyses were commonly used to check if a product belongs to a Protected Designation of Origin (PDO) or to a specific production area (Baroni et al., 2011; Camin et al., 2010; Di Paola-Naranjo et al., 2011; Magdas et al., 2016); but also to asses human health risks in public sanitary policies (Edelstein & Ben-Hur, 2018; Özbay, Dikici, & Soylukan, 2023). Given the rise in food fraud, food adulteration and environmental pollution, consumers are demanding more guarantees regarding the origin of food products. At the same time, more attention is paid to the quality of food and the overall diet, in a world where nutrition and health have become major concerns.

The geochemical signature of food products could be used as a marker of the connections between environmental conditions such as soil, plants, animals, water and atmospheric influences. In extensive agricultural practices dominated by grazing, trace and major element transfer from soil to vegetation and subsequently to animal tissues and secretions determined the multielemental composition of animal-derived foods (Drivelos & Georgiou, 2012). Profiling elemental composition is a powerful analytical approach for tracing geographical origin and authenticating the area of production of foods like edible oil, honey, wine and cheese (Drivelos & Georgiou, 2012; Luykx & van Ruth, 2008). This approach was frequently used to describe and differentiate the geographical origin of different food matrices such as wine (Di Paola-Naranjo et al., 2011), mushrooms (Nikkarinen & Mertanen, 2004), cereals (Podio et al., 2013), meat (Baroni et al., 2011), cheese (Moreno-Rojas, Sánchez-Segarra, Cámara-Martos, & Amaro-López, 2010; Suhaj & Koreňovská, 2008) and other foodstuffs (Baroni et al., 2015; Camin et al., 2010). However, its application to raw cow milk remained underexplored, particularly for authentication of the production area using trace element analysis.

In France, milk consumption reached approximately 49 kg per capita per year, making it an essential part of the diet (Agreste Graph'Agri, 2022). From a nutritional perspective, milk is a rich matrix, providing essential elements for the body's metabolism. The concentration of major elements in milk (Ca, K, P, Cl, Na, and Mg) has been extensively studied. Due to their nutritional benefits, the concentrations of major elements in milk were well documented, and numerous publications addressed these characteristics (Amalfitano et al., 2024; Gaucheron, 2005; Sibra, 2014; Tsioulpas, Lewis, & Grandison, 2007). The study of trace elements was mainly limited to specific areas, in particular the detection of contaminants present in high concentrations exceeding the maximum permitted levels and recognised as harmful to human health as well as the behaviour of heavy metals, such as lead (Pb), cadmium (Cd), copper (Cu) and mercury (Hg) (Enb, Donia, Abd-Rabou, Abou-Arab, & El-Senaity, 2009; Iqbal et al., 2020; Ribeiro Sant'Ana, de Carvalho, & da Silva, 2021).

Numerous studies characterized the inorganic content of milk, highlighting differences arising from sample preparation methods and variations in trace element concentrations across samples (Griboff, Baroni, Horacek, Wunderlin, & Monferran, 2019; Magdas et al., 2016; Özbay et al., 2023; Potočnik et al., 2020; Xu et al., 2021). Some trace elements in milks might present biological functions, such as chromium (Cr), cobalt (Co), manganese (Mn) and vanadium (V) (Mason, 2012; Nielsen, 2003). Arsenic (As) has been suggested to play a role in the activation of some enzymes or in the enhancement of the DNA synthesis, highlighting its potential biological significance. Other studies could differentiate geographical origin of milk from various sites based on the analysis of trace elements. Magdas et al. (2016) successfully discriminated the geographic origin of seventy seven milk samples from three distinct farms (Magdas et al., 2016). In this study, chromium (Cr), manganese (Mn), rubidium (Rb), strontium (Sr), and cesium (Cs) were identified as potential markers of the milk production area. Similarly, Xu et al. (2021) highlighted 15 elements (Tl, Sc, Nd, Eu, Gd, Dy, Sr, Ba, Mo, Rb, Cs, As, K, Ca) that could distinguish milks from New Zealand, Australia, and Austria (Xu et al., 2021). Finally, Potočnik et al. (2020) identified zinc (Zn), chlore (Cl), copper (Cu), manganese (Mn), selenium (Se), bromine (Br), rubidium (Rb), and strontium (Sr) as significantly discriminant between the Mediterranean, Pannonian, Alpine, and Dinaric regions (Potočnik et al., 2020). These studies showed that rubidium (Rb), strontium (Sr) and cesium (Cs) could be used as strong and reliable geochemical markers for tracing the geographic origin of milks. More recent studies combined multielement composition and stable isotopic ratios to authenticate geographical origin of dairy food products. At the same time they reinforced the comprehensive terroir-based conceptual framework (Danezis & Georgiou, 2022; Li et al., 2024; O' Sullivan, Cama-Moncunill, Salter-Townshend, Schmidt, & Monahan, 2023; O'Sullivan, Schmidt, & Monahan, 2022; Zhu et al., 2025). By using machine learning algorithms based on geo-isotopic and elemental composition, milk and dairy food geographical provenances could be more accurately determined (O' Sullivan et al., 2023). In that sense, the IsoFoodTrack database has recently been proposed for application to various food systems, including dairy based products, in order to align with authentication standards and to combat food fraud (Terro et al., 2025).

**Fig. 1:**
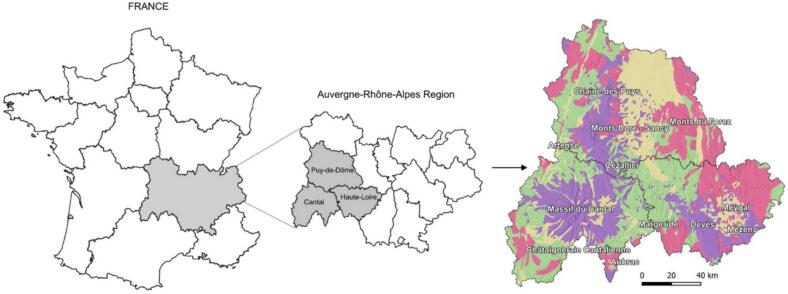
Map showing the location of the Auvergne-Rhone-Alpes region of France and the various mountain ranges (volcanic in violet, plutonic in pink, metamorphic in light green and sedimentary in yellow) that make up the departments of Puy-de-Dôme, Cantal and Haute-Loire. (For interpretation of the references to colour in this figure legend, the reader is referred to the web version of this article.)

Although milk does not benefit from a geographical indication scheme, which is reserved for processed products, it remains the raw material for fermented dairy products, such as cheeses, which in turn can be protected by a protected geographical indication (PGI) or by a Protected Designation of Origin (PDO). The concept of authenticating the geographical origin of this matrix is therefore particularly important, especially in regions where several PDOs cheeses coexist, as in the case of the French Massif central region. Massif central is a mountain pasture site shared by nine distinct PDO cheeses (Cantal, Salers, Saint Nectaire, Fourme d'Ambert and Bleu d'Auvergne, Fourme de Montbrison, Laguiole, Pélardon, Rocamadour). Massif central is characterized by a past intense volcanic activity through ages (from 7000 years ago to 12 million years ago) that led to different geological sites (Fig. 1). The testimony of volcanic eruptions at different times, shaped by glacial activity and currently undergoing erosion processes left pedogeochemical evidence in its morphology and lithological characteristics (Genevois et al., 2023; Nehlig et al., 2003). The combination of this unique geomorphology and varied volcanic lithology, generated specific climatic and pedogenic conditions (Legrand, Bartoli, & Curt, 2007; Quantin, 2004), which might influence the composition of certain elements in locally produced foodstuffs (Özbay et al., 2023; Sola-Larrañaga & Navarro-Blasco, 2009).

Recently, Coelho et al. (2025) showed the influence of volcanic conditions in the elemental and sensory characteristics of wines from PDO Côtes d'Auvergne in the Massif central region. To the best of our knowledge, no studies carried out on the elemental composition of milks or dairy products taking in account the volcanic parameter in the geographical authentication (Coelho et al., 2025).

However, previous studies have shown differences in texture and flavour between cheeses produced within the same Massif central region, depending on the production areas (Agabriel, Coulon, Journal, Sibra, & Albouy, 1999; Martin et al., 2009). The organoleptic diversity that characterises cheeses from this region is an advantage for consumers, who can choose products that suit their preferences. This richness may be influenced by the geographical location of the dairy farms and the geochemical signature given to the product. Thus, the production area and its environmental variables, such as the mountain range (geological age, climatology, lithology and pedology), may be involved in the chemical and organoleptic differentiation of products (Magdas et al., 2016; Sola-Larrañaga & Navarro-Blasco, 2009).

In this study, we characterized elemental composition of eighteen raw cow milks from the PDO Massif central cheeses production region. For that purpose, raw cow milks were sampled during the 2024 pasture season using morning milkings from single tanks. They originated from three distinct geographical sectors (Sancy, Cantal, Other) and two geological sites (volcanic vs non-volcanic) which were hypothesized to impart distinct multielemental compositions to the milks. The aim was to establish reference concentrations of trace elements for raw cow milks from different volcanic and non-volcanic sites. The objectives were: (i) to discriminate both geographical sectors (Sancy, Cantal, Other) and volcanic vs non-volcanic geological origins using ICP-MS-based ionomic profiling of raw cow milks from pasture farms in the French Massif central; and (ii) to assess the influence of dairy farms geological conditions (volcanic vs non-volcanic) on milk ionomic signatures. This approach could provide new insights into food traceability, product authenticity and the recognition of quality labels such as PDO cheeses.

## 2 Material and methods

### 2.1 Milk samples

**Fig. 2:**
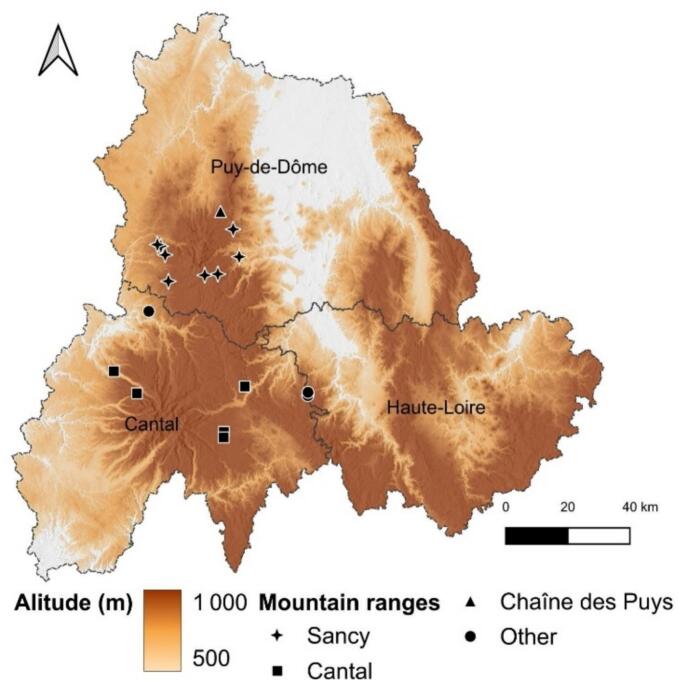
Map showing the location of milk sample collected: (■) farms located in Cantal mountain range (M-CA, *n* = 5); (✦) farms located in Sancy mountain range (M-SA, *n* = 8); (▲) farms located in Chaîne des Puys mountain range (M-CH, *n* = 1) and (●) farms located in non-volcanic area (M-T, *n* = 4).

Given the diversity in geographical areas, pedological and geological formations, climatic conditions and cheese-making procedures, it was essential that the farms selected reflected this diversity in order to ensure that the study area was as representative as possible. In collaboration with the interprofessional organizations of the Massif central PDO cheeses (Interprofession du Saint-Nectaire (ISN) and Comité Interprofessionnel des Fromages Cantal et Salers (CIF) and the Pôle Fromager AOP Massif central cheese cluster, specific criteria for choosing the farms were defined and forwarded to these institutions, which made a pre-selection of the affiliated farms. These criteria included the size of the plots and their homogeneity, the homogeneity of the herds in terms of breed, the use of extensive farming practices, maximizing grazing during the appropriate periods, and the selection of farms at different altitudes, scattered across different mountain ranges. These essential criteria further allowed the determination of geographical origin based on milk geochemical composition and the evaluation of its impact on the milk matrix (Table S.I.1.A). A total of eighteen raw cow milks (*N* = 18) were sampled from fifteen dairy and/or cheese farms spread across the Massif central in the departments of Puy-de-Dôme, Cantal and Haute-Loire and within the Cantal-Salers and Saint-Nectaire PDOs cheese production areas (Fig. 2). The names of the samples were coded according to their location within the different mountain ranges (Cantal = M-CA; Sancy: M-SA; Chaîne des Puys: M-CH; Control: M-T). A number was then added behind the start of each code to distinguish all the samples in the same area (M-SA1, M-SA2 …). Raw milks samples originated from three geographical origins: Sancy (SA, *N* = 8), Cantal (CA, *N* = 5) and Other mountains (OT, N = 5) and from two distinct geological origins: volcanic areas (V, *N* = 14) and non-volcanic areas (NV, *N* = 4). Cantal mountains are characterized by a volcanic lithology made of basalt and trachyandesite dating from 12 to 3 million years old. Sancy mountains exhibit a more recent volcanic lithology (ranging from 3 to 0.2 million years old) made of trachyte and phonolite. The investigated volcanic area from Chaîne des Puys present a more recent volcanic lithology (dating from 10,000 to 100,000 years old) made of basalts. Progressive erosion over time resulted in the formation to two different soil types, currently observed in these volcanic grazing areas: andosols (SA1, SA2, SA4, SA5, SA7, SA8 and CA2), and andic soils (SA3, SA6, CA1, CA3, CA4, CA5, OT5). Metamorphic lithologies dominated by gneiss occur in the Sumène Artense and Margeride regions where alocrisols are currently found (OT1, OT2, OT3 and OT4). Samples were collected between late April and mid-June 2024 directly from the farm's milk tank, just after milking in the morning. The milk collected was an average sample, taken from the herd that had grazed for at least 48 h on the same plot. For more information on the studied dairy farms, geographical with geological characteristics and zootechnical factors are indicated in Table S.I.1.B and Table S.I.1.C, respectively. A sterile container with a volume of one litre, supplied to the farmer in advance, was used for collection in order to prevent any external contamination. Milk samples were frozen and stored at −20 °C on the day of milking, prior to any analyses.

### 2.2 Analytical procedure

Milk samples were thawed before preparation in a clean room. Around 6 g of milk was sampled and placed in a PFA digestion Teflon vessel (Savillex, Eden Prairie, MN, USA). Samples were digested with 10 mL of ultra-pure nitric acid (HNO₃, 14 M) and 2 mL of hydrochloric acid (HCl, 6 M). Reagent grade nitric, and hydrochloric acids (Fluka, Seelze, Germany) were purified by sub boiling distillation in PFA DST-100 systems (Savillex, Eden Prairie, MN, USA). The tubes were placed in the MARS 6 microwave digestion system at 180 °C for 30 min. The resulting solution was transferred to a Teflon beaker and then subjected to evaporation on a hot plate at 70 °C. The solid residue was then taken up with 4 mL nitric acid (HNO₃, 7 M) and dried again at 70 °C. Finally, the residues were taken and diluted in 0.5 M HNO3–0.05 M HF acid mixture reaching a dilution factor of about 2. The solutions were directly transferred in 6 mL ICP-MS polystyrene vials, previously repeatedly washed with the same acid solution used for analysis. Vials were placed in a closed auto-sampler (SPS4) and trace element abundances were determined by Agilent 8900 ICP-MS Triple Quad (Agilent, Santa Clara, CA, USA). Sample solutions were aspired at a rate of 300 μL/min using a peristaltic pump and introduced into the plasma using a quartz introduction system (micromist nebulizer and Scott-type spray chamber). The analyses were performed in plasma robust mode with the RF forward power of 1550 W for all the experiments, and the interface was fitted with Ni sampling and skimmer cones designed for low poly-atomic formation. The reaction cell (He mode) was used to reduce interferences on masses ranging from 45Sc to 75As. The remaining elements were measured without operation of the collision cell and, thus, keeping their full sensitivity. The instrument was tuned so that the mass interferences of oxides and doubly charged ions (as monitored from CeO and Ce^2+^, respectively) do not exceed 1 % in both modes and thus can be neglected for most elements. The limit of quantification (LOQ) and limit of detection (LOD) were inferred for each measurement session from the reproducibility of the signal acquired on the ultra-clean acid mixture run every three samples. The LOQ end the LOD were calculated by adding to the average blank signal, 10 and 3 times the obtained standard deviation, respectively. The milk samples concentrations reported in this paper were systematically corrected for the total procedural blank contribution. The elements were measured using a method that consist of an uptake-stabilization time of 90s followed by a measurement sequence of ca. 300 s (including five sweeps from Li to U, with 3 points per mass and integration times ranging from 0.1 to 0.3 s depending on element first ionization energy), and 420 s of washing. Calibration curve for each element was performed by using several certified multi-elements solutions (CMS) ranging from 0.001 to 100 ppb (i.e. 0.001, 0.01, 0.1, 0.5, 1, 10 and 100 ppb). All these solutions were prepared by gravimetric dilution of 10 μg/mL certified solutions traceable to NIST (Inorganic Ventures, Virginia, USA). R2 values for linear calibration curves were between 0.99 and 1.00 for all elements.

### 2.3 Statistical analysis

For the statistical analysis of milks from different mountain areas, only three groups of samples were statistically analyzed and compared: samples from the Cantal (M-CA; N = 5), samples from the Sancy (M-SA; N = 8), and samples from the non-volcanic area (M-T; N = 4). Sample from the Chaîne des Puys (M-CH1) was not included in the analysis of geographical origin but was integrated in the analysis of geological origin, permitting to compare milks from volcanic (N = 14) and non-volcanic areas (N = 4). The concentrations of milk elements were tested for normal distribution using the Shapiro-Wilk test. The Levene's test was subsequently applied to assess the homogeneity of variances between the two investigated factors: geography (comprising three groups CA, SA and OT) and geology (comprising two groups V and NV). As fewer than 10 % of the elements satisfied two tests, the multielement data was therefore analyzed using non-parametric statistical methods. Specifically, Kruskal-Wallis test was applied followed by a Wilcoxon post-hoc tests with Holm-Bonferroni correction, on each milk element, with the objectives to differentiate the three geographical origins and the two geological areas. Interaction between geology and geography factors was assessed by an aligned rank transform test. A *p*-value <0.05 was considered for significant differences. Boxplots were created using the ggplot2 package and the ggplot function in R software for data visualization. In order to take a global look at the database generated from the ICP-MS data, a Principal Component Analysis (PCA) analysis was carried out on R software with the FactoMineR package using the entire multielement dataset of *N* = 18 milks. Pearson correlation analysis was performed on the elemental concentrations to assess linear relationships and to identify significant co-variations relevant to geochemical processes.

## 3 Results and discussion

### 3.1 Concentration of trace elements in pasture raw cow milks

Of the 61 elements analyzed, only 32 were detected and quantified in the milk samples. As shown in Table 1 and Table S.I.2, the concentrations of trace elements measured in all raw cow milks were of the same order of magnitude as those found in literature (Benincasa, Lewis, Sindona, & Tagarelli, 2008; Griboff et al., 2019; Magdas et al., 2016; Özbay et al., 2023; Potočnik et al., 2020; Sola-Larrañaga & Navarro-Blasco, 2009; Xu et al., 2021; Zwierzchowski & Ametaj, 2018). None of the quantified elements exceeded the maximum concentration limits set by current regulations for dairy products, foodstuffs or drinking water (“(EU) 2023/915,”, 2023; Group, 1993; Zwierzchowski & Ametaj, 2018).

**Table 1:** Mean and standard deviation of concentration of trace elements (in μg/kg) in sample collected and comparisons with maximum permitted levels in dairy products or drinking water. Concentration ranges from literature data are also given (Benincasa et al., 2008; Griboff et al., 2019; Magdas et al., 2016; Özbay et al., 2023; Potočnik et al., 2020; Sola-Larrañaga & Navarro-Blasco, 2009; Xu et al., 2021; Zwierzchowski & Ametaj, 2018). The following elements are not explicitly presented here: Ag, Be, Bi, Ce, Dy, Er, Eu, Ga, Gd, Ge, Hf, Hg, Ho, In, La, Lu, Nd, Pr, Sm, Ta, Tb, Tm and Yb because they are below the detection and/or quantification limits of the device for all the milks. The “NF” values indicate that no maximum/allowable/Oral MLR concentration has been established for this element to date. The “nd” values indicate undetermined values. For Oral MRLs (Minimal Risk Levels) the following letter a, b, c refer to the duration of the exposition to the contaminant and its concentration: a = acute (1–14 days); b = intermediate (14 and 364 days); c = chronic (1 year or more).

Elements	Range concentration in raw cow milk of our study - (μg/kg)	Mean ± Standard Deviation of element concentrations in raw cow milk of our study - (μg/kg)	Range concentration in raw cow milk from literature^1^ - (μg/kg)	Maximum permitted concentrations in foodstuff in Europe (2023) - μg/kg	World Health Organization -allowable limits in drinking water - μg/kg	Oral MLR (ATSDR, 2024) - μg/kg/day
Li	0.061–0.361	0.171 ± 0.092	0.2–337	NF	NF	NF
B	11.80–32.87	22.69 ± 5.66	90.8–470	NF	2400	200
Sc	0.002–0.038	0.025 ± 0.011	4–232	NF	NF	NF
Ti	4.69–39.63	9.53 ± 7.68	47–2190	NF	NF	NF
V	0.001–0.369	0.036 ± 0.084	1.2–33.6	NF	NF	0.1c
Cr	0.001–0.403	0.130 ± 0.126	0.2–5332	NF	50	NF
Mn	2.07–8.84	5.03 ± 1.78	6.8–435,800	NF	80	NF
Fe	35.2–179.32	52.0 ± 32.6	55.4–33,500	NF	NF	NF
Co	0.042–0.104	0.062 ± 0.018	0.72–116	NF	NF	20b
Ni	0.018–0.275	0.058 ± 0.064	4–4590	NF	70	NF
Cu	3.58–13.18	8.72 ± 2.70	6.9–3260	NF	2000	20b
Zn	421–868.35	741 ± 98	1107–3.48E+6	NF	NF	NF
As	0.004–0.119	0.045 ± 0.034	0.02–223	10*	10	0.3c
Rb	151–2996.39	883 ± 677	114–33,870	NF	NF	NF
Sr	37.2–153.16	90.6 ± 32.6	7–7020	NF	NF	2000b
Y	0.001–0.036	0.005 ± 0.008	2.3–3.7	NF	NF	NF
Zr	0.002–0.522	0.066 ± 0.127	NF	NF	NF	NF
Nb	0.0006–0.139	0.012 ± 0.033	NF	NF	NF	NF
Mo	4.62–13.42	9.28 ± 2.31	29–290	NF	NF	60b
Pd	0.020–0.087	0.050 ± 0.019	NF	NF	NF	NF
Cd	0.004–0.013	0.008 ± 0.002	0.01–7.3	10	3	0.1c
Sn	0.002–0.046	0.019 ± 0.014	1.2–19.2	50,000	NF	300b
Sb	0.001–0.014	0.004 ± 0.004	nd-11.3	NF	20	0.6b
Te	0.010–0.101	0.034 ± 0.023	0.21–0.47	NF	NF	NF
Cs	0.296–5.933	1.74 ± 1.39	0.24–1080	NF	NF	NF
Ba	11.78–37.69	21.56 ± 7.75	34.8–1793	NF	1300	NF
W	0.001–0.121	0.033 ± 0.038	nd−1.8	NF	NF	NF
Pb	0.008–0.062	0.029 ± 0.016	0.03–220	20	10	NF
Th	2.3E-05 - 0.020	0.002 ± 0.005	nd–2.3	NF	NF	NF
U	0.0001–0.0050	0.001 ± 0.001	nd-2.4	NF	30	0.2c
Au	0.0003–0.0341	0.013 ± 0.009	0.42–3.94	NF	NF	NF
Tl	9.1E-05 - 0.0724	0.012 ± 0.016	nd-3.6	NF	NF	NF

**Fig. 3:**
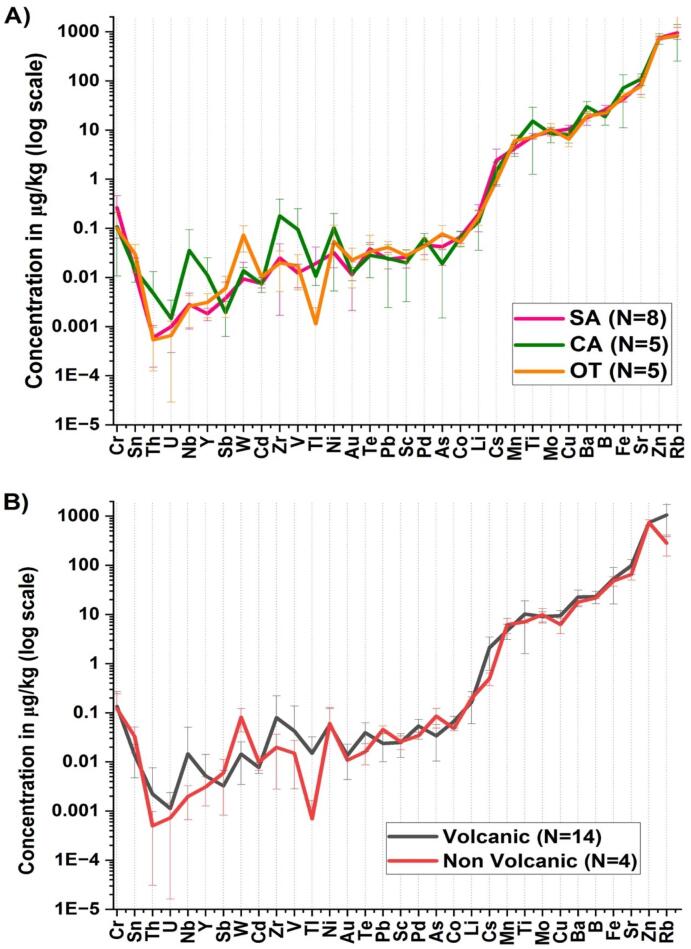
Elemental profiles of milk samples represented by their mean concentrations displayed on a logarithmic scale according to their geographic provenance (A) and geological origin (B). Error bars represent the standard deviation. Milk samples are classified as originating from SA (Sancy), CA (Cantal) and OT (Other) mountain areas and from volcanic and non-volcanic grazing areas. The following elements are not explicitly presented here: Ag, Be, Bi, Ce, Dy, Er, Eu, Ga, Gd, Ge, Hf, Hg, Ho, In, La, Lu, Nd, Pr, Sm, Ta, Tb, Tm and Yb because they are below the detection and/or quantification limits of the device for all the milk samples.

The elements identified in the samples, in order of decreasing average concentration, were Rb, Zn, Sr, Fe, B, Ba, Ti, Mo, Cu, Mn, Cs, Li, Cr, Zr, Co, Ni, Pd, As, V, Te, W, Pb, Sc, Sn, Au, Tl, Nb, Cd, Y, Sb, Th, and U (Fig. 3). In all analyzed samples, Ag, Be, Bi, Ce, Dy, Er, Eu, Ga, Gd, Ge, Hf, Hg, Ho, In, La, Lu, Nd, Pr, Sm, Ta, Tb, Tm and Yb were below the detection and/or quantification limits of the analytical instrument. As, B, Ba, Cd, Co, Cs, Cu, Fe, Li, Mn, Mo, Ni, Pd, Pb, Rb, Sb, Sr, Te, Ti, Tl, U, V, Y, Zr and Zn were detected in all samples at concentrations above the instrumental detection and quantification limits. Au, Cr, Nb, Sc, Sn, Th and W were not detected or quantified (below LOD or below LOQ) in the following samples: M-SA7, M-SA8, M-T2, M-T3 and M-T2T3 for Au; M-SA1, M-SA3, M-SA5, M-SA6, M-SA7 and M-SA8 for Cr; M-T1 or Nb, M-SA8 and M-CA3 for Sc; M-SA1, M-SA7 and M-SA8 for Sn; M-SA6 for Th and M-SA2, M-SA4, M-SA7 and M-SA8 for W.

The analyzed raw cow milks multielements belong to the groups of alkali metals, alkaline earth metals, other metals, transition metals, common metalloids and actinides. Mean concentrations and standard deviations of raw cow milk elements are presented on a logarithmic scale, according to their geographic provenance (Fig. 3A) and geological origin (Fig. 3B). A wide range of concentrations was observed, ranging from 1.10^−5^ to 1.10^3^ μg/kg. On average, the elements with concentrations above 1 μg/kg were distributed as follows, in descending order Rb > Zn > Sr > Fe > B > Ba> Ti > Mo > Cu > Mn > Cs with Rb and Zn concentrations close to the mg/kg. The elements with the lowest concentrations, with an average of less than 0.01 μg/kg, were Cd > Y > Sb > Th > U. Some elements showed a wider dispersion of concentrations, reflecting greater heterogeneity between samples for these specific elements.

Coefficients of variation of element concentrations varied from 13 % to 267 %. Zn, Mo, B, Co, Cd had inter-sample coefficients of variation of less than 30 % and were ranked in increasing order. On the other hand, Sn, Rb, Cs, Ti, Cr, Sb, U, Ni, W, Tl, Y, Zr, V, Th and Nb showed inter-sample coefficients of variation exceeding 75 %, indicating marked heterogeneity and the possible influence of external factors on their concentrations. Comparison of the trace element concentrations obtained in this study with literature revealed significant differences for some elements. In particular, Sc, Y, Ni, Au, Co, B, V, Mo, Te and Sn concentrations were quantified by a factor comprised between 2 and 100 lower than those reported in other studies (Benincasa et al., 2008; Griboff et al., 2019; Magdas et al., 2016; Özbay et al., 2023; Potočnik et al., 2020; Sola-Larrañaga & Navarro-Blasco, 2009; Xu et al., 2021; Zwierzchowski & Ametaj, 2018). Such differences could reflect differences based on milk geographical provenance. To our knowledge, in contrast to previous studies, our study enabled for the first time the quantification of Pd, Zr and Nb in raw cow milks. On the other hand, the concentrations of Li, Ti, Cr, Fe, Mn, Cu, Zn, As, Rb, Sr, Cd, Sb, Cs, Ba, W, Pb, Th, U and Tl remained comparable to values reported in the scientific literature. The diversity found in the multielemental composition of raw cow milks from Massif central was further analyzed in order to check its ability to describe geographical and geological origins.

### 3.2 Geographical vs geological distinction of pasture raw cow milks

**Fig. 4:**
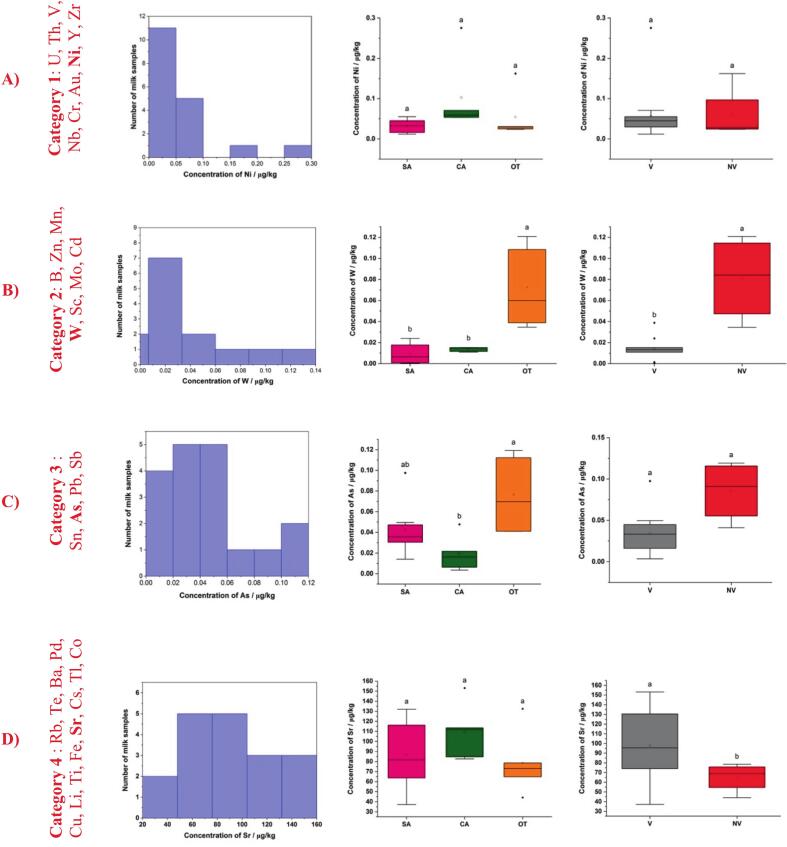
Examples of data distribution and mean comparisons of elemental concentration between mountain ranges (SA, CA and OT, respectively for Sancy, Cantal and Other) and geological origin (V and NV for Volcanic and Non-Volcanic, respectively) for elements Ni (A), W (B), As (C) and Sr (D). Each of these four elements belongs to a different category, according to their ability to distinguish or not geographical and/or geological origins (p < 0.05): category 1 (elements with no differences among geography and geology factors), category 2 (elements distinguishing both geography and geology factors), category 3 (elements differentiating the sole geography factor) and category 4 (elements differentiating the sole geology factor). Different letters indicate statistically significant differences (p < 0.05).

The thirty-two trace elements analyzed in raw cow milks could be statically classified into four categories according to their ability to distinguish or not geographical and/or geological origins (*p* < 0.05). Fig. 4 illustrates those four categories, and the results of the non-parametric statistical tests are presented in Table S.I.3. Nine milk elements from category 1 (U, Th, V, Nb, Cr, Au, Ni, Y, Zr) presented no statistical differences among geographical provenances and geology origins among the *N* = 18 milks studied (Fig. 4A). Seven milk elements from category 2 (B, Zn, Mn, W, Sc, Mo, Cd) permitted to distinguish both geography and geology factors (Fig. 4B). In category 3, four elements (Sn, As, Pb, Sb) could distinguish milks based on their geographical provenance (Fig. 4C) and in category 4, twelve elements (Rb, Te, Ba, Pd, Cu, Li, Ti, Fe, Sr, Cs, Tl, Co) could discriminate milks according to their geological origins (Fig. 4D).

Significant interaction between geographical and geological origin was observed for four elements: Rb, Te, Ba and B meaning that the geographical origin distinction in milks with these four elements also depends also on their geological origin (Table S.I.3). For all the other statistically differentiating elements, the geographical could be distinguished regardless of the geological origin and vice versa. However, those statistical results should be interpreted with caution, given the moderate number of milk samples and the non-uniform distribution of geographical and geological factors.

The geographical origin was further compared by pairs (Sancy/Cantal, Sancy/Other and Cantal/Other) based on Kruskal-Wallis tests, followed by post-hoc comparisons using the Wilcoxon test with Holm-Bonferroni adjustment (Table S.I.4). Table S.I.5 presents the mean concentration values and standard deviations of each analyzed elements for the three geographical origins. When comparing Sancy vs Cantal milks from the Cantal mountains had significantly higher mean concentrations in four elements (Ni, Y, Nb and Ba). In the pair comparison Sancy/Other, milks from Sancy mountains were characterized by higher mean concentration in four elements (Cu, Cs and Tl) and lower mean concentration in one element (W) compared to ‘Other’ mountains. Finally, when comparing Cantal/Other, milks from Cantal were characterized by higher concentration in two elements (Nb and Tl) and lower mean concentration in two elements (As and W) compared to milks from Other mountains. Concerning the geological origin influence on milks, six elements (Cu, Rb, Pd, Te, Cs, Tl) were found at significantly higher levels in volcanic areas, whereas three elements (W, As and Pb) were significantly higher in non-volcanic areas (Table S.I.6).

**Fig. 5:**
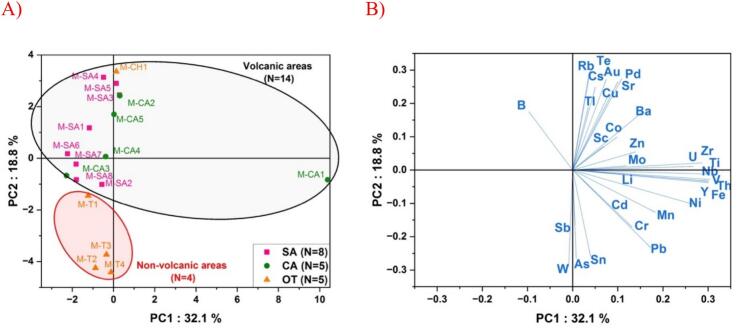
Scatter biplot of the first two principal components resulting from the first Principal Component Analysis (PCA) of the whole milk multielement dataset (*N* = 18), showing milk sample scores (A) and the inorganic element loadings (B). Geographical provenance is symbolized by squares for Sancy mountains, circles for Cantal mountains and triangles for Other moutains. Geological provenances are circled in red for non-volcanic areas and in black for volcanic areas. (For interpretation of the references to colour in this figure legend, the reader is referred to the web version of this article.)

In order to visualize the multielemental diversity among the eighteen raw cow milks originating from three distinct geographical sites (CA, SA, Other) and two geological sites (V and NV), a principal component analysis (PCA) was performed on the whole milk multielemental concentrations dataset to interpret the variability of elemental concentrations among the milk samples. The resulting biplot on the two first principal components depicts the projections of individuals (milk samples) (Fig. 5A) and variables (multielement concentrations) (Fig. 5B).

The two first components of the first PCA analysis permitted to explain 50.9 % of the total variance of all milks sampled during the 2024 pasture season. PCA provided an integrative overview of the ability of ionomics to differentiate geographical provenances and geological origins (Fig. 5A). The first principal component PC1 could explain 32.1 % of the entire dataset and was largely driven by M-CA1 multielemental composition, with elevated concentrations of U, Z, Ti, Nb, V, Th, Y, Fe and Ni. (Fig. 5B). This PC1 axis tended to distinguish milks originating from Cantal mountains vs Sancy/Other mountains (except for milk M-CA3). Milks originating from Sancy mountains were grouped for lower values of PC1 and driven by differences in milk elemental composition in B, Rb, Cs, Te, Au, Tl, Cu, Pd and Sr and Ba. The second principal component PC2, explaining 18.8 % of the entire dataset could be used to discriminate geological provenances of grazing areas (volcanic vs non-volcanic), regardless of their geographical provenances. All the *N* = 14 raw cow milks originating from volcanic sites located in the Cantal, Sancy and Chaîne des Puys exhibited higher values along the PC2 axis, dominated by the loadings: Rb, Te, Cs, Tl, Au, Cu, Pd, Sr, B and Ba. All the *N* = 4 raw cow milks originating from non-volcanic grazing areas were associated with lower PC2 scores and driven by the loadings Sb, W, As and Sn. Our findings align with previous studies showing that elements such as Rb, Sr, and Cs, Tl, As can be used to determine geographical origin of milks (Magdas et al., 2016; Xu et al., 2021). Even if our study, only focused on the sole variability of grazing areas during one pasture season, ionomics can highlight elemental distributions of Rb, Te, Cs, Tl, Cu Pd, Sr and Ba, enabling to authenticate geological provenances of raw cow milks, regardless of its geographical origins. Those results are, as far as we know, novel in the field of tracking geological history on animal grazing areas in dairy production systems. The integration of geo-isotopic methods with ionomics, as recently proposed (Li et al., 2024; O' Sullivan et al., 2023; Zhu et al., 2025), would provide a more thorough evaluation of geological influence within dairy systems, while also considering potential cofounding parameters such as seasonality, lactation stage, feeding regimen and interannual variability.

### 3.3 Relationships between multielements unravelling the importance of geological origin in multielement partioning in raw cow milks

**Fig. 6:**
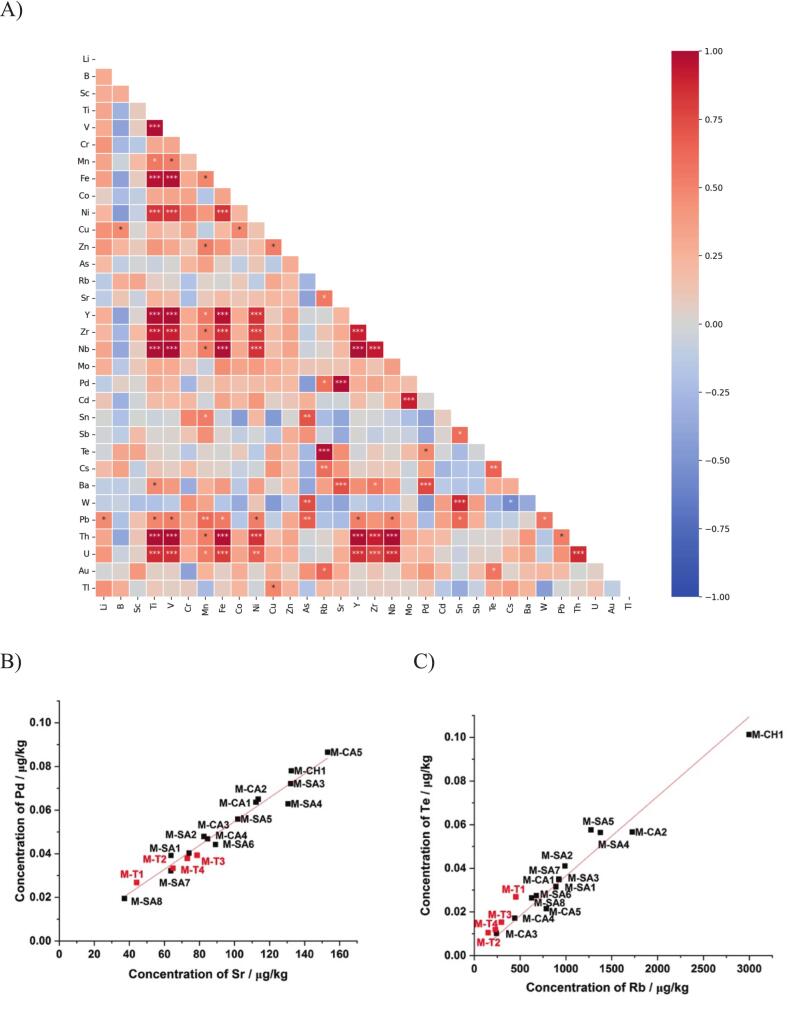
Pearson correlation plot between the entire dataset of multielement content in raw cow milks from Massif central. Red Colour indicates positive correlations, and blue colour indicates negative correlations. Symbols *, ** and *** indicate the significance of the correlations, characterized with *p*-value <0,05, <0.01 and < 0.001, respectively (A). Biplot of elemental concentrations of Pd vs Sr (B) and Te vs Rb (C) for all the *N* = 18 milks that are labelled. Red symbols represent the *N* = 4 milks from non-volcanic areas and black symbols represent the *N* = 14 raw cow milks originating from volcanic areas. Red lines show the good linear regressions passing through the origin. (For interpretation of the references to colour in this figure legend, the reader is referred to the web version of this article.)

We further performed a Pearson correlation analysis on the concentrations of the thirty-two elements across the eighteen raw cow milks in order to evaluate the relationships between raw cow milk elements, regardless of the geographical and geological origin (Fig. 6A). However, it should be mentioned that, due to non-uniform distribution of samples, the observed correlations were more likely attributable to volcanic traits than to non-volcanic ones. All elements, except Sc and Cr, presented at minimum one pair combination with an associated Pearson correlation coefficient (r) that was significant (*p* < 0.05). Most of element pairs exhibited positive correlations, except for the couple (W/Cs) (*r* = −0.55, p < 0.05). Some elements, such as Ti, were involved in up to eleven element pairs with significant positive correlation coefficients (Ti/U, Ti/Th, Ti/Pb, Ti/Ba, Ti/Nb, Ti/Zr, Ti/Y, Ti/Ni, Ti/Fe, Ti/Mn, Ti/V). Other elements were involved in only a single pair, such as (Mo/Cd), exhibiting a significant positive correlation. All these combinations suggest that similar biogeochemical processes govern the presence of these elements in raw cow milks. The element pairs (Sr/Pd) and (Rb/Te) exhibited in this study the highest Pearson correlation coefficients and the highest levels of significance among the entire datasets. Fig. 6B and C illustrate the very strong correlation of two element pairs across the entire milk dataset, regardless of geological and geographical considerations. Interestingly, the (Sr/Pd) and (Rb/Te) element pairs accumulated to a greater extent in milks originating from volcanic areas than in milks from non-volcanic areas. Those elements may reveal similar biogeochemical transfers from bedrocks weathering to soil and into raw cow milks, mainly controlled by the farm's geological environment, regardless of geographical context. The two elements Sr and Pd, together with Fe and Ti have been reported in literature as typical geomarker of alkali basalts found in the ancient volcanoes of Massif central (Briot, 1990; Chauvel & Jahn, 1984; Nehlig et al., 2003). The two other elements Rb and Te, presenting similar significant strong correlations, highlight the importance of considering the specific geological context of the volcanic areas to explain soil/milk transfers in the future.

Although the sampled dairy farms are geographically close, lithological differences persist between the areas studied, which may influence the variability of trace element concentrations in milk. This observation suggests a possible transfer of specific elements from volcanic soils to milk via pasture-based cattle feeding. Some sets of elements found in raw cow milks, particularly Sr, Pd, Rb and Te support the concept of terroir at the trace element level. Their accumulation in milks along geology-driven pathways could discriminate raw cow milks from different geological areas, as illustrated by the Massif central PDO cheeses regions. To confirm and characterize such phenomenon more precisely, it would be necessary to analyse the concentrations of trace elements in the soil and in the plants of the plot grazed by animals. A comparison between the geochemical profiles of bioavailable soils, plant covers and milks would make possible to validate the influence of the local lithology on the elemental composition of raw cow milks. These ongoing studies in our research group will complement our findings on geochemical transfers into milks from volcanic areas.

The proposed exploratory study, conducted during the 2024 single pasture season using morning milkings from single tank, will need to be expanded in the future by incorporating a larger number of samples that capture greater variability – particularly in terms of seasonality, lactation stage, feeding regimen, and interannual variation. Such strategy is expected to increase the statistical power of the findings and to support the development of a more generalized framework for using ionomics on raw cow milks to trace geographical and geological origins.

## 4 Conclusion

This study explored milk ionome and demonstrated that ICP-MS signatures provided a robust tool for characterizing the multielemental composition of raw cow milks in mountain pasture systems. Ionomics could also be used to differentiate milks from areas that are geographically close but differ in geological origins.

Our study established the elemental profiles of thirty-two elements in eighteen raw cow milk samples from the PDO Massif central cheese-producing region. Some elements such as Pd, Zr or Nb were reported, to our knowledge, for the first time in milk matrices. Multivariate statistical analysis permitted to discuss the ability of milk multielements to differentiate geographical provenances (Sancy, Cantal and Other mountains) and geological origins (volcanic and non-volcanic lithologies). In that sense, eleven elements (B, Zn, As, Pb, Mn, Sn, W, Sc, Sb, Mo, and Cd) and nineteen elements (Rb, Te, Ba, B, Zn, Pd, Cu, Li, Ti, Fe, Sr, Cs, Mn, Tl, W, Sc, Mo, Cd, and Co) could discriminate geographical and geological origins, respectively. The Pearson correlations analyzed among milk elements revealed unprecedented territorial signatures linked to their geological environment, emphasizing the scientific basis of the concept of terroir in dairy production. Specific sets of elements – particularly (Rb,Te) and (Sr,Pd) - were strongly correlated in all milk samples, suggesting environmentally driven soils to milk transfer processes, with higher accumulation in milks from volcanic areas.

Overall, this study opens new perspectives for using milk ionomics as a complementary approach to assess food traceability, authenticity, and the typicity of mountain dairy products. Our exploratory study highlights the need to consider more cofounding factors such as season, lactation stage, feeding regimen and interannual variability. These factors should be accounted for before generalizing the use of ionomics to unravel the geographical and geological origins of raw cow milks in a single region, as shown in the Massif central PDO cheese region. Finally, expanding the study to include more milk samples over multiple years would strengthen the robustness of our results. Further studies should also focus on the soil-plant-milk chain to provide evidence of chemical transfers and support the concept of milk terroir for the PDO cheese community.

## CRediT authorship contribution statement

**Camille Martin:** Writing – original draft, Methodology, Investigation, Formal analysis, Data curation, Conceptualization. **Abdelmouhcine Gannoun:** Writing – original draft, Supervision, Methodology, Formal analysis, Data curation. **Christophe Poix:** Writing – review & editing. **Laurent Rios:** Writing – review & editing, Conceptualization. **Christian Coelho:** Writing – review & editing, Writing – original draft, Funding acquisition, Formal analysis, Data curation, Conceptualization.

## Ethical statement

Milk samples were collected from farms with the informed consent of the farmers and the prior agreement of the interprofessional organizations of the Massif central PDO cheeses (Interprofession du Saint-Nectaire (ISN) and Comité Interprofessionnel des Fromages Cantal et Salers (CIF) and the Pôle Fromager AOP Massif central cheese cluster. No experimental procedures were performed on animals, and sample collection did not interfere with routine farming practices.

## Funding sources

This work was supported by Isite CIR1 Axe 4, Clermont Auvergne Métropole and 10.13039/501100011073VetAgro Sup.

## Declaration of competing interest

The authors declare that they have no known competing financial interests or personal relationships that could have appeared to influence the work reported in this paper.

## Data Availability

Data will be made available on request.
