# Supplementary material for: Ionomic exploration of the geographical and geological origins of mountain pasture cow milks in the French Massif central
Source: Food Chem X. 2026 Jan 5;33:103501. doi: 10.1016/j.fochx.2026.103501 (PMC12816871; doi:10.1016/j.fochx.2026.103501)
Supplement: Supplementary material [file mmc1.docx]

# IONOMIC EXPLORATION OF THE GEOGRAPHICAL AND GEOLOGICAL ORIGINS OF MOUNTAIN PASTURE COW MILKS IN THE FRENCH MASSIF CENTRAL

Camille Martin^1^, Abdelmouhcine Gannoun^2^, Christophe Poix^1^, Laurent Rios^1^, Christian Coelho^1,*^

1 Université Clermont Auvergne, INRAE, VetAgro Sup campus agronomique de Lempdes, UMR F, F-15000 Aurillac, France
2 Laboratoire Magmas et Volcans, Université Clermont Auvergne, CNRS, IRD, OPGC, Clermont-Ferrand, France

*corresponding author : christian.coelho@vetagro-sup.fr

**Supplementary Information**

**Table S.I.1** (A) Criteria used to select the farms included in the study and the plots chosen for milk monitoring

| Type of criteria | Criteria 1 | Criteria 2 | | Criteria 3 |
| --- | --- | --- | --- | --- |
| Criteria used to select farms in the Massif Central that illustrate the region's diversity | | | | |
| Mountain range | SANCY | CANTAL | | OTHER |
| Diversity of altitude range | < 1000 m |  | | > 1075 m |
| Slope | East | | West | |
| Lithology | Volcanic | | Non-volcanic | |
| Shared criteria between farms used to select the plot monitored | Plot large enough for animals to graze for several days | | | |
|  | Homogeneous plot in terms of its geological. pedological. topographical and botanical characteristics. | | | |

**Table S.I.1** (B) Geographical and geological **characteristics of the studied dairy farms**

| Milk samples | GPS Lambert coordinates (X/Y) | Geography (mountains) | Geology origin | lithology | soils |
| --- | --- | --- | --- | --- | --- |
| M-SA1 | 676565.167083 / 6486989.713225 | Sancy | Volcanic | Trachyte/  Phonolite | Andosol |
| M-SA2 | 675525.154420 / 6495458.109530 | Sancy | Volcanic | Trachyte/  Phonolite | Andosol |
| M-SA3 | 699395.1435150276 / 6494809.503916233 | Sancy | Volcanic | Trachyte/  Phonolite | Andic soil |
| M-SA4 | 688280.580352 / 6488927.906531 | Sancy | Volcanic | Trachyte/  Phonolite | Andosol |
| M-SA5 | 692515.157272 / 6489290.565946 | Sancy | Volcanic | Trachyte/  Phonolite | Andosol |
| M-SA6 | 697458.945861 / 6503599.378438 | Sancy | Volcanic | Trachyte/  Phonolite | Andic soil |
| M-SA7 | 674110.668642 / 6498192.847243 | Sancy | Volcanic | Trachyte/  Phonolite | Andosol |
| M-SA8 | 673003.384695 / 6498705.671963 | Sancy | Volcanic | Trachyte/  Phonolite | Andosol |
| M-CA1 | 658985.994473 / 6458219.467776 | Cantal | Volcanic | Basalt | Andic soil |
| M-CA2 | 666416.888739 / 6451167.89423 | Cantal | Volcanic | Basalt | Andosol |
| M-CA3 | 694475.239104 / 6438882.463823 | Cantal | Volcanic | Basalt | Andic soil |
| M-CA4 | 694381.785592 / 6437109.7861420 | Cantal | Volcanic | Basalt | Andic soil |
| M-CA5 | 701214.327380 / 6453394.394271 | Cantal | Volcanic | Basalt | Andic soil |
| M-OT1 | 670246.725927 / 6477385.088407 | Other  (Sumène-Artense) | Non-Volcanic | Metamorphic/  Gneiss | Alocrisol |
| M-OT2 | 721535.4717386416 / 6450475.153259133 | Other  (Margeride) | Non-Volcanic | Metamorphic/  Gneiss | Alocrisol |
| M-OT3 | 721600.663462 / 6451498.878162 | Other  (Margeride) | Non-Volcanic | Metamorphic/  Gneiss | Alocrisol |
| M-OT4 | 721535.4717386416 / 6450475.153259133 | Other  (Margeride) | Non-Volcanic | Metamorphic/  Gneiss | Alocrisol |
| M-OT5 | 693367.27402 / 6509315.112649 | Other  (Chaîne des Puys) | Volcanic | Basalt | Andic soil |

**Table S.I.1** (C) Animal and farm factors in the **studied dairy farms**

| Farm | Breed composition | Extra Regimen Feeding | Water sources |
| --- | --- | --- | --- |
| SA1 | Montbeliarde, Holstein | Barley, Hay, Corn | Tap water |
| SA2 | Montbeliarde, Holstein, Jersiaise, Brune des Alpes, Abondance | Hay, Corn, VL18 supplement | Tap water |
| SA3 | Montbeliarde, Abondance, Ferrandaise | Hay, Corn, Lucerne, VL18 supplement | Tap water |
| SA4 | Montbeliarde, Abondance, Ferrandaise | Hay, Corn, Lucerne, VL18 supplement | Tap water |
| SA5 | Montbeliarde, Holstein | Barley, Hay, Corn | Tap water |
| SA6 | Montbeliarde | Hay, VL18 supplement | Tap water |
| SA7 | Holstein | Hay, VL18 supplement | Tap water |
| SA8 | Salers | Hay, VL18 supplement | Tap water |
| CA1 | Salers | Hay, VL18 supplement | Tap water |
| CA2 | Salers | Hay, VL18 supplement | Tap water |
| CA3 | Holstein | Hay, Corn, Silage | Tap water |
| CA4 | Montbéliarde | Hay, Silage, Corn | Tap water |
| CA5 | Salers | Hay, Corn | Tap water |
| OT1 | Holstein | Hay, Corn, Colza | Tap water |
| OT2 | Abondance, Holstein, Jersiaise | Hay, Lucerne, Corn | Tap water |
| OT3 | Abondance, Holstein, Jersiaise | Hay, Lucerne, Corn | Tap water |
| OT4 | Abondance, Holstein, Jersiaise | Hay, Lucerne, Corn | Tap water |
| OT5 | Abondance, Montbeliarde | Hay, Barley, wheat, lucerne | Tap water |

**Table S.I.2** Concentration of trace elements (in µg/kg) in raw cow milks collected for the studies (N=18). Limit of Detection (LOD) and Limit of quantification (LOQ) are indicated for each elements (in µg/kg)

| **Elements** | **M-SA1** | **M-SA2** | **M-SA3** | **M-SA4** | **M-SA5** | **M-SA6** | **M-SA7** | **M-SA8** | **M-CA1** | **M-CA2** | **M-CA3** | **M-CA4** | **M-CA5** | **M-T1** | **M-T2** | **M-T3** | **M-T4** | **M-CH1** | **LOD** | **LOQ** |
| --- | --- | --- | --- | --- | --- | --- | --- | --- | --- | --- | --- | --- | --- | --- | --- | --- | --- | --- | --- | --- |
| **Li** | 0.3610 | 0.1445 | 0.1785 | 0.2047 | 0.3482 | 0.1368 | 0.0686 | 0.1016 | 0.2975 | 0.0893 | 0.0638 | 0.1636 | 0.0612 | 0.1960 | 0.1756 | 0.2154 | 0.1933 | 0.0731 | *0.02097* | *0.06990* |
| **B** | 29.2256 | 16.3119 | 25.3701 | 32.8685 | 28.7048 | 29.5465 | 18.9112 | 26.0639 | 14.2293 | 27.6090 | 11.8015 | 18.8119 | 20.3506 | 20.9565 | 21.9848 | 21.2284 | 22.4282 | 22.0179 | *0.07945* | *0.26483* |
| **Sc** | 0.0380 | 0.0249 | 0.0319 | 0.0131* | 0.0285 | 0.0347 | 0.0107 | nd | 0.0295 | 0.0379 | nd | nd | 0.0098* | 0.0322 | 0.0238 | 0.0315 | 0.0152* | 0.0353 | *0.00733* | *0.02443* |
| **Ti** | 7.0752 | 7.0417 | 8.2888 | 7.5455 | 8.6877 | 6.4100 | 6.1770 | 8.7282 | 39.6349 | 11.4346 | 4.6926 | 9.7234 | 10.3574 | 7.4520 | 7.3448 | 7.0895 | 6.5065 | 7.4084 | *0.04982* | *0.16607* |
| **V** | 0.0077 | 0.0116 | 0.0131 | nf | nd | 0.0096 | 0.0199 | 0.0178 | 0.3692 | 0.0268 | 0.0159 | 0.0374 | 0.0214 | 0.0069 | 0.0317 | 0.0168 | 0.0049* | 0.0256 | *0.00187* | *0.00623* |
| **Cr** | nd | 0.4034 | nd | 0.1210 | nd | nd | nd | nd | 0.2726 | 0.0649 | 0.0599* | 0.1200 | 0.0251* | nd | 0.1393 | 0.0564* | 0.2836 | nd | *0.01801* | *0.06003* |
| **Mn** | 5.1250 | 5.0400 | 3.8151 | 5.4364 | 3.5224 | 3.4886 | 2.9059 | 4.3427 | 8.5925 | 6.2814 | 2.0650 | 5.2622 | 4.3433 | 4.0533 | 6.8758 | 8.8426 | 4.9744 | 5.4932 | *0.01580* | *0.05267* |
| **Fe** | 40.0697 | 43.4264 | 51.7077 | 36.0328 | 49.4463 | 35.9873 | 45.1058 | 38.9478 | 179.3231 | 39.2909 | 36.3114 | 51.1503 | 52.1071 | 60.2205 | 35.1563 | 51.3644 | 45.4385 | 45.4624 | *0.08500* | *0.28333* |
| **Co** | 0.0511 | 0.0650 | 0.1035 | 0.0429 | 0.0730 | 0.0594 | 0.0702 | 0.0704 | 0.0838 | 0.0447 | 0.0599 | 0.0917 | 0.0449 | 0.0466 | 0.0515 | 0.0419 | 0.0519 | 0.0709 | *0.00175* | *0.00582* |
| **Ni** | 0.0171 | 0.0550 | 0.0465 | 0.0318 | 0.0335 | 0.0117 | 0.0148 | 0.0434 | 0.2754 | 0.0533 | 0.0706 | 0.0532 | 0.0590 | 0.0250 | 0.0238 | 0.0303 | 0.1625 | 0.0295 | *0.00306* | *0.01021* |
| **Cu** | 12.3176 | 8.1581 | 13.1805 | 10.5564 | 11.8069 | 7.3238 | 9.2055 | 11.0033 | 10.6347 | 8.7364 | 3.5751 | 9.3556 | 8.3035 | 7.0187 | 4.1405 | 4.8523 | 8.8608 | 7.8938 | *0.00428* | *0.01428* |
| **Zn** | 758.6008 | 750.9024 | 777.6622 | 713.2775 | 815.2658 | 693.9272 | 749.6409 | 727.5723 | 861.9125 | 789.5626 | 420.8982 | 774.1589 | 801.6560 | 750.7089 | 631.2042 | 868.3465 | 724.5921 | 720.5774 | *0.02661* | *0.08870* |
| **As** | 0.0496 | 0.0333 | 0.0449 | 0.0141 | 0.0333 | 0.0276 | 0.0975 | 0.0381 | 0.0477 | 0.0162 | 0.0035 | 0.0218 | 0.0063 | 0.0411 | 0.0697 | 0.1192 | 0.1122 | 0.0410 | *0.00249* | *0.00829* |
| **Rb** | 890.2638 | 989.4215 | 890.6269 | 1375.1717 | 1273.4851 | 677.2675 | 925.4038 | 625.2825 | 921.3140 | 1724.3473 | 242.5829 | 441.3737 | 785.9638 | 453.5077 | 150.9310 | 295.4243 | 230.7738 | 2996.3872 | *0.00049* | *0.00163* |
| **Sr** | 63.7488 | 74.0323 | 132.0300 | 130.5574 | 101.8898 | 89.1637 | 63.6633 | 37.1812 | 112.1840 | 113.5207 | 82.6088 | 84.6174 | 153.1555 | 44.1870 | 64.8915 | 78.6426 | 73.0700 | 132.4356 | *0.00620* | *0.02067* |
| **Y** | 0.0008 | 0.0015 | 0.0024 | 0.0022 | 0.0023 | 0.0018 | 0.0018 | 0.0018 | 0.0359 | 0.0045 | 0.0029 | 0.0063 | 0.0059 | 0.0011 | 0.0053 | 0.0038 | 0.0022 | 0.0031 | *0.00011* | *0.00037* |
| **Zr** | 0.0072 | 0.0241 | 0.0730 | 0.0132 | 0.0463 | 0.0126 | 0.0086 | 0.0139 | 0.5216 | 0.2515 | 0.0362 | 0.0483 | 0.0335 | 0.0017 | 0.0197 | 0.0151 | 0.0424 | 0.0201 | *0.00019* | *0.00062* |
| **Nb** | 0.0007* | 0.0014 | 0.0037 | 0.0055 | 0.0052 | 0.0009* | 0.0015 | 0.0037 | 0.1391 | 0.0128* | 0.0024 | 0.0133 | 0.0093 | nd | 0.0032 | 0.0021 | 0.0006* | 0.0045 | *0.00045* | *0.00150* |
| **Mo** | 8.5387 | 8.9282 | 11.8710 | 8.2537 | 10.2604 | 9.0089 | 8.1923 | 8.1974 | 12.5057 | 4.6190 | 7.3751 | 7.7862 | 9.6836 | 13.4199 | 7.1316 | 7.2745 | 11.8982 | 12.1086 | *0.00075* | *0.00249* |
| **Pd** | 0.0392 | 0.0403 | 0.0721 | 0.0629 | 0.0559 | 0.0442 | 0.0322 | 0.0195 | 0.0636 | 0.0650 | 0.0479 | 0.0468 | 0.0865 | 0.0268 | 0.0333 | 0.0393 | 0.0379 | 0.0780 | *0.00278* | *0.00925* |
| **Cd** | 0.0064 | 0.0088 | 0.0098 | 0.0057 | 0.0082 | 0.0071 | 0.0067 | 0.0072 | 0.0103 | 0.0038 | 0.0066 | 0.0074 | 0.0088 | 0.0125 | 0.0078 | 0.0065 | 0.0129 | 0.0108 | *0.00066* | *0.00222* |
| **Sn** | nd | 0.0339 | nd | 0.0109* | 0.0072* | 0.0076* | nd | nd | 0.0183 | 0.0221 | 0.0126* | 0.0053* | 0.0134 | 0.0063* | 0.0420 | 0.0384 | 0.0454 | 0.0170 | *0.00418* | *0.01392* |
| **Sb** | 0.0013* | 0.0073 | 0.0010* | 0.0010* | 0.0036 | nd | 0.0023 | 0.0128 | 0.0041 | 0.0013* | 0.0008* | 0.0013* | 0.0022 | 0.0026 | 0.0046 | 0.0136 | 0.0031 | 0.0060 | *0.00068* | *0.00228* |
| **Te** | 0.0317 | 0.0411 | 0.0315 | 0.0564 | 0.0576 | 0.0275 | 0.0348 | 0.0264 | 0.0351 | 0.0566 | 0.0102 | 0.0172 | 0.0215 | 0.0269 | 0.0105 | 0.0153 | 0.0119 | 0.1013 | *0.00000* | *0.00000* |
| **Cs** | 2.3019 | 3.1081 | 1.0268 | 5.9326 | 2.7177 | 0.7767 | 2.3645 | 0.9567 | 2.3068 | 2.1632 | 0.6291 | 0.9765 | 1.4198 | 0.5834 | 0.2956 | 0.6158 | 0.4942 | 2.6959 | *0.00025* | *0.00082* |
| **Ba** | 14.8381 | 14.1500 | 19.0946 | 26.3116 | 25.6579 | 11.7819 | 16.7225 | 13.8801 | 33.1726 | 35.2274 | 25.1725 | 18.2111 | 37.6928 | 16.9820 | 15.4729 | 22.0808 | 16.8154 | 24.7935 | *0.00207* | *0.00690* |
| **W** | nd | nd | 0.0115* | nd | nd | 0.0240* | nd | nd | 0.0153* | 0.0154* | 0.0116* | 0.0150* | 0.0109* | 0.0346 | 0.1084 | 0.0600 | 0.1208 | 0.0388 | *0.01096* | *0.03653* |
| **Pb** | 0.0321 | 0.0235 | 0.0177 | 0.0199 | 0.0310 | 0.0104 | 0.0351 | 0.0178 | 0.0622 | 0.0225 | 0.0115 | 0.0082 | 0.0171 | 0.0351 | 0.0541 | 0.0448 | 0.0483 | 0.0229 | *0.00028* | *0.00094* |
| **Th** | 0.0002 | 0.0014 | 0.0008 | 0.0003 | 0.0009 | nd | 0.0002* | 0.0004 | 0.0198 | 0.0014 | 0.0004 | 0.0013 | 0.0011 | nd | 0.0009 | 0.0009 | 0.0002* | 0.0007 | *0.00008* | *0.00028* |
| **U** | 0.0005 | 0.0003 | 0.0017 | 0.0022 | 0.0011 | 0.0007 | 0.0002 | 0.0013 | 0.0050 | 0.0006 | 0.0004 | 0.0008 | 0.0005 | 0.0001* | 0.0016 | 0.0010 | 0.0002 | 0.0004 | *0.00007* | *0.00024* |
| **Au** | 0.0208 | 0.0044 | 0.0240 | 0.0092* | 0.0094* | nd | nd | nd | 0.0148* | 0.0172* | 0.0116* | 0.0097* | 0.0081* | 0.0109* | nd | nd | nd | 0.0341 | *0.00598* | *0.01994* |
| **Tl** | 0.0124 | 0.0066 | 0.0108 | 0.0163 | 0.0724 | nd | 0.0200 | 0.0147 | 0.0155 | 0.0116 | 0.0065 | 0.0071 | 0.0122 | 0.0021* | nd | nd | nd | 0.0029 | *0.00073* | *0.00244* |

*nd for not detected, meaning the element has not been detected (content below LOD). ~~with or its content was below the LOQ).~~ For elements Sc, V, Cr, Nb, Sn, Sb, W, U, Th Au, Tl, concentrations with the symbol * were measured between LOD and LOQ. Their interpretation should be taken cautiously.*

**Table S.I.3.** Statistical results of the Kruskal Wallis test followed by the Wilcoxon Holm–Bonferroni post hoc test for geographical and geological distinction for the 32 elements. Interaction between geography and geology is done with the aligned rank transform test. Values in bold present statistically significant differences (p<0.05).

|  | **Geographical origin** | **Geological origin** | **Geography x Geology** |
| --- | --- | --- | --- |
| **Rb** | 0.584 | **<0.001** | **<0.001** |
| **Te** | 0.305 | **<0.001** | **0.001** |
| **Ba** | 0.051 | **<0.001** | **0.010** |
| **B** | **0.0033** | **<0.001** | **0.040** |
| **Zr** | 0.633 | 0.101 | 0.094 |
| **Zn** | **<0.001** | **<0.001** | 0.096 |
| **Pd** | 0.173 | **<0.001** | 0.111 |
| **Y** | 0.982 | 0.182 | 0.126 |
| **Ni** | 0.437 | 0.211 | 0.137 |
| **Cu** | 0.343 | **<0.001** | 0.149 |
| **As** | **<0.001** | 0.407 | 0.154 |
| **Au** | 0.068 | 0.491 | 0.157 |
| **Li** | 0.079 | **0.042** | 0.166 |
| **Ti** | 0.725 | **0.024** | 0.207 |
| **Cr** | 0.690 | 0.145 | 0.216 |
| **Nb** | 0.712 | 0.270 | 0.242 |
| **V** | 0.840 | 0.252 | 0.253 |
| **Fe** | 0.285 | **0.026** | 0.281 |
| **Pb** | **<0.001** | 0.752 | 0.309 |
| **Sr** | 0.178 | **<0.001** | 0.311 |
| **Th** | 0.733 | 0.253 | 0.330 |
| **Cs** | 0.528 | **0.001** | 0.368 |
| **Mn** | **<0.001** | **0.028** | 0.381 |
| **Tl** | 0.319 | **0.018** | 0.579 |
| **Sn** | **0.003** | 0.572 | 0.589 |
| **W** | **<0.001** | **0.013** | 0.639 |
| **U** | 0.962 | 0.085 | 0.651 |
| **Sc** | **0.037** | **0.037** | 0.706 |
| **Sb** | **0.040** | 0.968 | 0.727 |
| **Mo** | **<0.001** | **<0.001** | 0.839 |
| **Cd** | **<0.001** | **0.009** | 0.943 |
| **Co** | 0.126 | **<0.001** | 0.944 |

**Table S.I.4** Statistical results of the Kruskal-Wallis tests followed by the Wilcoxon Holm-Bonferroni post hoc tests on mean comparisons of elemental concentrations between mountain ranges. Bold values with the symbol * are significant (p ≤ 0.05).

| **Elements** | **Kruskal-Wallis test  (p-value)** | **Post hoc test Wilcoxon and Holm-bonferroni  (p-value adjusted)** | | |
| --- | --- | --- | --- | --- |
|  |  | **Sancy vs. Cantal** | **Sancy vs. Other** | **Cantal vs. Other** |
| Li | 0.3583 | 0.3203 | 0.9215 | 0.3203 |
| B | 0.0702 | 0.0657 | 0.2863 | 0.2863 |
| Sc | 0.6844 | 0.7202 | 0.8456 | 0.7202 |
| Ti | 0.1541 | 0.1359 | 0.8309 | 0.1359 |
| V | 0.0574 | 0.0540 | 0.5542 | 0.1646 |
| Cr | 0.3035 | 0.3117 | 0.3117 | 0.7257 |
| Mn | 0.1472 | 0.3315 | 0.1799 | 0.5536 |
| Fe | 0.4000 | 0.4925 | 0.4925 | 0.8127 |
| Co | 0.4541 | 0.8053 | 0.5614 | 0.5614 |
| **Ni** | **0.0324*** | **0.0393*** | 0.8566 | 0.0572 |
| **Cu** | **0.0368*** | 0.2604 | **0.0326*** | 0.2604 |
| Zn | 0.4983 | 0.5363 | 0.7425 | 0.5363 |
| **As** | **0.0242*** | 0.1504 | 0.1504 | **0.0192*** |
| Rb | 0.1876 | 0.4021 | 0.2046 | 0.4021 |
| Sr | 0.2517 | 0.2552 | 0.7363 | 0.2552 |
| **Y** | **0.0072*** | **0.0052*** | 0.1652 | 0.1652 |
| **Zr** | **0.0424*** | 0.0572 | 0.9738 | 0.0572 |
| **Nb** | **0.0394*** | **0.0466*** | 0.8082 | **0.0466*** |
| Mo | 0.6633 | 0.7301 | 0.7301 | 0.7301 |
| Pd | 0.1150 | 0.1407 | 0.6221 | 0.1407 |
| Cd | 0.2217 | 0.8533 | 0.2736 | 0.2736 |
| Sn | 0.1791 | 0.4795 | 0.1980 | 0.3868 |
| Sb | 0.1137 | 0.6212 | 0.1394 | 0.1394 |
| Te | 0.2258 | 0.3408 | 0.3276 | 0.7223 |
| **Cs** | **0.0500** | 0.3283 | **0.0461*** | 0.2888 |
| **Ba** | **0.0362*** | **0.0334*** | 0.5709 | 0.1133 |
| **W** | **0.0090*** | 0.5212 | **0.0131*** | **0.0287*** |
| Pb | 0.0675 | 0.6190 | 0.0856 | 0.0856 |
| Th | 0.0809 | 0.0832 | 0.7929 | 0.0832 |
| U | 0.5224 | 0.9214 | 0.5603 | 0.5603 |
| Au | 0.4193 | 0.5622 | 0.5622 | 0.5622 |
| **Tl** | **0.0093*** | 0.5764 | **0.0084*** | **0.0426*** |

**Table S.I.5** Mean and standard deviation of trace element concentrations of milks from the Cantal (N=5), the Sancy (N=8) and Other moutain (N=5). Letters in brackets refer to the mean comparisions followed by a post-hoc Dunn test comparing geographical origin for each element. When letters differ, geographical differences are significant (p<0.05)

| **Mean ± SD Element concentrations** | **Units** | **Geographical origin** | | | |
| --- | --- | --- | --- | --- | --- |
|  |  | ***Sancy (N=8)*** | | ***Cantal (N=5)*** | ***Other (N=5)*** |
| **Li** | µg/kg | 0.193 ± 0.1082 (a) | 0.1351 ± 0.0998 (a) | | 0.1707 ± 0.0563 (a) |
| **B** | µg/kg | 25.8753 ± 5.6298 (a) | 18.5605 ± 6.1153 (a) | | 21.7232 ± 0.6093 (a) |
| **Sc** | µg/kg | 0.026 ± 0.0105 (a) | 0.0198 ± 0.0166 (a) | | 0.0276 ± 0.0081 (a) |
| **Ti** | µg/kg | 7.4943 ± 0.9908 (a) | 15.1686 ± 13.9202 (a) | | 7.1602 ± 0.3915 (a) |
| **V** | µg/kg | 0.0124 ± 0.0062 (a) | 0.0941 ± 0.154 (a) | | 0.0172 ± 0.0116 (a) |
| **Cr** | µg/kg | 0.2622 ± 0.1997 (a) | 0.1085 ± 0.0978 (a) | | 0.0989 ± 0.1165 (a) |
| **Mn** | µg/kg | 4.2095 ± 0.9179 (a) | 5.3089 ± 2.4076 (a) | | 6.0479 ± 1.8664 (a) |
| **Fe** | µg/kg | 42.5905 ± 5.8965 (a) | 71.6366 ± 60.6044 (a) | | 47.5284 ± 9.1857 (a) |
| **Co** | µg/kg | 0.0669 ± 0.0181 (a) | 0.065 ± 0.0218 (a) | | 0.0526 ± 0.011 (a) |
| **Ni** | µg/kg | 0.0317 ± 0.016 (b) | 0.1023 ± 0.097 (a) | | 0.0542 ± 0.0606 (ab) |
| **Cu** | µg/kg | 10.444 ± 2.059 (b) | 8.1211 ± 2.6889 (ab) | | 6.5532 ± 2.0033 (a) |
| **Zn** | µg/kg | 748.356 ± 37.936 (a) | 729.638 ± 175.767 (a) | | 739.0858 ± 85.1976 (a) |
| **As** | µg/kg | 0.0423 ± 0.0248 (ab) | 0.0191 ± 0.0176 (a) | | 0.0766 ± 0.0376 (b) |
| **Rb** | µg/kg | 955.865 ± 260.279 (a) | 823.116 ± 571.399 (a) | | 825.405 ± 1218.693 (a) |
| **Sr** | µg/kg | 86.533 ± 33.562 (a) | 109.217 ± 28.597 (a) | | 78.6453 ± 32.7911 (a) |
| **Y** | µg/kg | 0.0018 ± 0.0005 (b) | 0.0111 ± 0.0139 (a) | | 0.0031 ± 0.0016 (ab) |
| **Zr** | µg/kg | 0.0249 ± 0.0232 (a) | 0.1782 ± 0.2129 (a) | | 0.0198 ± 0.0147 (a) |
| **Nb** | µg/kg | 0.0028 ± 0.0019 (a) | 0.0354 ± 0.0581 (a) | | 0.0026 ± 0.0017 (a) |
| **Mo** | µg/kg | 9.1563 ± 1.2932 (a) | 8.3939 ± 2.9252 (a) | | 10.3666 ± 2.9466 (a) |
| **Pd** | µg/kg | 0.0458 ± 0.0171 (a) | 0.062 ± 0.0161 (a) | | 0.0431 ± 0.0201 (a) |
| **Cd** | µg/kg | 0.0075 ± 0.0014 (a) | 0.0074 ± 0.0024 (a) | | 0.0101 ± 0.0028 (a) |
| **Sn** | µg/kg | 0.0124 ± 0.0124 (a) | 0.0143 ± 0.0064 (a) | | 0.0298 ± 0.0172 (a) |
| **Sb** | µg/kg | 0.0037 ± 0.0043 (a) | 0.0019 ± 0.0013 (a) | | 0.006 ± 0.0045 (a) |
| **Te** | µg/kg | 0.0384 ± 0.0124 (a) | 0.0281 ± 0.0183 (a) | | 0.0332 ± 0.0386 (a) |
| **Cs** | µg/kg | 2.3981 ± 1.6774 (b) | 1.4991 ± 0.7297 (ab) | | 0.937 ± 0.9911 (a) |
| **Ba** | µg/kg | 17.8046 ± 5.4865 (b) | 29.8953 ± 8.0436 (a) | | 19.2289 ± 4.0025 (ab) |
| **W** | µg/kg | 0.0093 ± 0.011 (b) | 0.0136 ± 0.0022 (ab) | | 0.0725 ± 0.0398 (a) |
| **Pb** | µg/kg | 0.0234 ± 0.0086 (a) | 0.0243 ± 0.0219 (a) | | 0.041 ± 0.0123 (a) |
| **Th** | µg/kg | 0.0006 ± 0.0005 (a) | 0.0048 ± 0.0084 (a) | | 0.0005 ± 0.0004 (a) |
| **U** | µg/kg | 0.001 ± 0.0007 (a) | 0.0015 ± 0.002 (a) | | 0.0007 ± 0.0006 (a) |
| **Au** | µg/kg | 0.0114 ± 0.0093 (a) | 0.0123 ± 0.0037 (a) | | 0.0225 ± 0.0164 (a) |
| **Tl** | µg/kg | 0.0192 ± 0.0223 (b) | 0.0106 ± 0.0038 (ab) | | 0.0011 ± 0.0013 (a) |

**Table S.I.6** Mean and standard deviation of trace element concentrations of milks from the volcanic areas (N=14) and non-volcanic areas (N=4). Letters in brackets refer to the mean comparisions followed by a post-hoc Dunn test comparing geographical origin for each element. When letters differ, geographical differences are significant (p<0.05).

| **Mean ± SD Element concentrations** | **Units** | **Geological origin** | |
| --- | --- | --- | --- |
|  |  | **Volcanic areas (N=14, Sancy, Cantal, Chaîne des puys)** | **Non-volcanic areas (N=4, Other)** |
| **Li** | µg/kg | 0.164 ± 0.104 (a) | 0.195 ± 0.016 (a) |
| **B** | µg/kg | 22.99 ± 6.43 (a) | 21.65 ± 0.678 (a) |
| **Sc** | µg/kg | 0.025 ± 0.012 (a) | 0.026 ± 0.008 (a) |
| **Ti** | µg/kg | 10.23 ± 8.65 (a) | 7.10 ± 0.42 (a) |
| **V** | µg/kg | 0.043 ± 0.094 (a) | 0.015 ± 0.012 (a) |
| **Cr** | µg/kg | 0.135 ± 0.136 (a) | 0.120 ± 0.123 (a) |
| **Mn** | µg/kg | 4.69 ± 1.61 (a) | 6.19 ± 2.13 (a) |
| **Fe** | µg/kg | 53.17 ± 36.79 (a) | 48.04 ± 10.52 (a) |
| **Co** | µg/kg | 0.067 ± 0.018 (a) | 0.048 ± 0.005 (a) |
| **Ni** | µg/kg | 0.057 ± 0.065 (a) | 0.060 ± 0.068 (a) |
| **Cu** | µg/kg | **9.43 ± 2.45 (b)** | 6.22 ± 2.15 (a) |
| **Zn** | µg/kg | 739.69 ± 101.95 (a) | 743.72 ± 97.65 (a) |
| **As** | µg/kg | 0.034 ± 0.024 (a) | **0.086 ± 0.037 (b)** |
| **Rb** | µg/kg | **1054.21 ± 673.49 (b)** | 282.667 ± 128.32 (a) |
| **Sr** | µg/kg | 97.91 ± 32.84 (a) | 65.20 ± 15.10 (a) |
| **Y** | µg/kg | 0.0052 ± 0.0090 (a) | 0.0031 ± 0.0018 (a) |
| **Zr** | µg/kg | 0.079 ± 0.142 (a) | 0.020 ± 0.017 (a) |
| **Nb** | µg/kg | 0.015 ± 0.036 (a) | 0.0020 ± 0.0013 (a) |
| **Mo** | µg/kg | 9.09 ± 2.10 (a) | 9.93 ± 3.21 (a) |
| **Pd** | µg/kg | **0.054 ± 0.019 (b)** | 0.034 ± 0.006 (a) |
| **Cd** | µg/kg | 0.0077 ± 0.0019 (a) | 0.0099 ± 0.0032 (a) |
| **Sn** | µg/kg | 0.014 ± 0.009 (a) | 0.033 ± 0.018 (a) |
| **Sb** | µg/kg | 0.0032 ± 0.0034 (a) | 0.0060 ± 0.0052 (a) |
| **Te** | µg/kg | **0.039 ± 0.023 (b)** | 0.016 ± 0.007 (a) |
| **Cs** | µg/kg | **2.10 ± 1.38 (b)** | 0.497 ± 0.144 (a) |
| **Ba** | µg/kg | 22.62 ± 8.43 (a) | 17.84 ± 2.91 (a) |
| **W** | µg/kg | 0.0141 ± 0.011 (a) | **0.081 ± 0.041 (b)** |
| **Pb** | µg/kg | 0.024 ± 0.014 (a) | **0.046 ± 0.008 (b)** |
| **Th** | µg/kg | 0.0022 ± 0.0053 (a) | 0.0005 ± 0.0005 (a) |
| **U** | µg/kg | 0.0011 ± 0.0013 (a) | 0.0007 ± 0.0007 (a) |
| **Au** | µg/kg | 0.014 ± 0.009 (a) | 0.011 ± 0.008 (a) |
| **Tl** | µg/kg | **0.015 ± 0.017 (b)** | 0.0007 ± 0.0010 (a) |
